# Teaching for Student and Societal Wellbeing in HPE: Nine Pedagogies for Social Justice

**DOI:** 10.3389/fspor.2021.702922

**Published:** 2021-08-11

**Authors:** Göran Gerdin, Rod Philpot, Wayne Smith, Katarina Schenker, Kjersti Mordal Moen, Lena Larsson, Susanne Linnér, Knut Westlie

**Affiliations:** ^1^Department of Sport Science, Faculty of Social Sciences, Linnaeus University, Växjö/Kalmar, Sweden; ^2^Faculty of Education and Social Work, School of Curriculum and Pedagogy, The University of Auckland, Auckland, New Zealand; ^3^Department of Public Health and Sport Sciences, Faculty of Social and Health Sciences, Inland Norway University of Applied Sciences, Elverum, Norway

**Keywords:** wellbeing, health, physical education, social justice, pedagogy

## Abstract

We currently find ourselves living in precarious times, with old and new social inequities on the rise due to the challenges associated with an unprecedented rise of global migration and neoliberalism, amplified in our post COVID-19 world. Research has demonstrated that there is a high correlation between inequality at the societal level and the overall health and wellbeing of individuals within those societies. We believe that school health and physical education (HPE) has a significant role to play in addressing and acting on social inequities that impact on the wellbeing of both students and society as a whole. Based on the findings of an international research project called EDUHEALTH which explored pedagogies for social justice in school health and physical education (HPE) across Sweden, Norway and New Zealand, this paper aims to highlight the addressing of (in)equality and student wellbeing through HPE practice. In particular, the paper presents nine different but complementary pedagogies for social justice that we believe can improve individual, collective, and societal wellbeing. We conclude by proposing that, if adopted across a whole school curriculum, these nine pedagogies for social justice could form the basis of a holistic school-wide community approach aimed at improving both student and societal wellbeing.

## Introduction

We currently find ourselves living in precarious times (Kirk, 2020), with old and new social inequities on the rise due to the challenges associated with an unprecedented rise of global migration and neoliberalism, amplified in our post COVID-19 world. Wilkinson and Pickett (2010) demonstrate in multiple ways that societal inequities correlate strongly with a wide range of social problems (e.g., drug use, violence, imprisonment, educational performance) and negative health outcomes (mental health, obesity, life expectancy). In addition to disproportionally affecting indigenous communities and marginalized groups within society, Wilkinson and Pickett (2010) argue that “the effects of inequality are not confined just to the least well off: instead, they affect the vast majority of the population” (p. 176).

The challenge of addressing these inequities is immense, requiring action at a global level through organizations such as the United Nations and The World Health Organization (WHO); at the national level through government policies in regard to labor, education and health, and at the local and individual level through community movements and individual action. Within education, the subject of Health and Physical Education (HPE) has been positioned by many as best placed to support the health and wellbeing of young people (O'Sullivan, 2004). Thirty years ago, Sallis and McKenzie (1991) argued that HPE should be considered one of key public entities that addresses public health. This argument draws strength from research that highlights the positive role that physical activity plays in reducing chronic diseases such as coronary heart disease, type II diabetes, osteoporosis, and some forms of cancer (Dishman et al., 2004; Bouchard et al., 2007). Perhaps as a response to the pressure from public health advocates who suggest that HPE has *not* picked up on public health goals (e.g., McKenzie and Lounsbery, 2009; Sallis et al., 2012), Kirk (2018) recently suggested that the leading justification for HPE is to take responsibility for health and wellbeing.

We whole-heartedly agree about the important role that HPE can play in addressing health and wellbeing inequities. Our point of departure is a move from positioning health as something to be achieved, something “normal,” and individual and an outcome of the physical activity within HPE, to a concept that recognizes that health as a relation between the individual and the surroundings environment that is influenced both positively and negatively by multiple causal factors (Quennerstedt, 2008). This salutogenic approach to health (Antonovsky, 1996) positions health not as something you have or do not have, but rather as a complex continuum that acknowledges various starting points, environmental impacts, and that lifestyles of young people are not simply a matter of “good” or “bad” life choices. The focus of a salutogenic approach to health acknowledges public health goals but also advocates that self-understanding, joy of movement, expression, and empowering experiences are also health promoting (McCaughtry and Rovegno, 2001). This conception of health aligns with the WHO Ottawa Charter for WHO (1986) which states that health promotion is the process of enabling people to increase control over and to improve, their health. To reach “a state of complete physical, mental, and social wellbeing” (WHO, 1986), an individual/group must be able to identify and realize aspirations, satisfy needs and change or cope with the environment.

Building on these aims and the scholarship within HPE (e.g., Quennerstedt, 2008; Webb et al., 2008; McCuaig and Quennerstedt, 2018) we are proposing that HPE has a significant role to play in attaining health and wellbeing goals. HPE can do this by providing meaningful and pleasurable experiences of movement, physical activity, and sport for all students, regardless of their ability, gender, sexuality, ethnicity or cultural, and socio-economic backgrounds. As Hallal et al. (2006) and Perkins et al. (2004) state, when young people enjoy movement and physical activity in contexts such as games, sport, and HPE, movement and physical activity, these activities are more likely to become a natural part of their lives and something they enjoy and carry on with throughout their adulthood. Notably in relation to pedagogical work in classrooms, the role of the teacher shifts from making student healthy to a dialectical responsibility to prepare students with resources needed to be healthy while preparing a learning environment that supports health. Promoting and striving for equitable health outcomes in and through school HPE is therefore about providing students with experiences in various movement contexts that develop their abilities and skills to take critical action (Wright, 2004), both by themselves and with others, that is, critical action which is underpinned by the values of inclusion, democracy, and social justice.

Returning to Wilkinson and Pickett (2010), they argue that since humans are social beings, the quality of our relationships with one another is integral to human wellbeing. They conclude that inequities in society lead to breakdowns in trust and cooperation and increases in conflict and social exclusion. Therefore, they call for the creation of a society that encourages' mutual interdependence and co-operation, in which each person's security depends on the quality of their relationships with others and [where] a sense of self-worth comes less from status than from the contribution made to the wellbeing of others' (Wilkinson and Pickett, 2010, p. 210). We agree with this and believe that school and HPE are uniquely placed to cultivate such a society.

Based on the findings of an international research project called EDUHEALTH which explored pedagogies for social justice in HPE across Sweden, Norway, and New Zealand, this paper aims to highlight the addressing of (in)equality and wellbeing through HPE practice. In particular, the paper will focus on the improvement of individual, collective and societal wellbeing through use of nine different but complementary pedagogies for social justice. We conclude by calling for a holistic school approach to addressing wellbeing where HPE together with other school subjects all play an important part.

## Well-Being, Pedagogies for Social Justice, and Physical Education

The basic premise for the practice of school HPE is that it can and does contribute to physically active, healthy, and socially responsible citizens (Doll-Tepper and Scoretz, 2001; UNESCO, 2015; WHO, 2018) who in turn contribute to the wellbeing of society as a whole. HPE as a learning area contributes significantly to holistic school wellbeing, encompassing promotion of health and wellbeing on individual, relational, and collective wellbeing (Morgan and Bourke, 2008; Opstoel et al., 2020). Indeed, many HPE curricula around the world now include a strong focus on health and wellbeing with curriculum documents moving from having the title physical education (PE) to HPE (e.g., in Australia, New Zealand, and Sweden) while in other countries, such as Scotland, physical education is part of a larger curriculum area called “Health and Well-being.” The HPE curriculum in New Zealand, for instance, expects that the students should learn to: “…contribute to the wellbeing of those around them, of their communities, of their environments (including natural environments), and of the wider society” (2007, p. 22). HPE is further claimed to improve the students' physical, social, affective, and cognitive abilities although there is also a recognition that these outcomes are highly dependent on contextual and pedagogic variables (Bailey et al., 2009).

In addition, some HPE curricula are underpinned by a socially-critical perspective, meaning that teaching and learning should foster *active* and *critical* consumers of physical activity (Macdonald and Tinning, 2003; Goodyear et al., 2019). As a focus on socially-critical perspectives has become more prevalent in HPE curricula, there has been a corresponding growth in the adoption of socially-critical and social justice pedagogies in classroom-based health contexts, perhaps more than the traditional teaching spaces of physical educators. Enactment of social justice perspectives in and through the physical in PE classes remains a challenge for many HPE teachers, despite the growth of critical health education in the classroom. A study by McIntyre et al. (2016) that explored New Zealand secondary school HPE teachers' understandings and use of social justice pedagogies reported that these practices were conceived as health education rather than physical education pedagogies. It appears for many teachers it is more difficult to teach both for and about social justice in the gymnasium and on the sport field than it is in a classroom-based lesson (Scorringe et al., 2021). In an Australian context, a recent case study by Alfrey and O'Connor (2020) reported on how a large HPE department in Australia worked alongside a group of researchers to transform their secondary HPE to align with the critical intentions of the Australian HPE curriculum.

It has been argued that pedagogies for social justice should question assumptions about *power* and *social relations* (Shelley and McCuaig, 2018). They should also be concerned with political and economic factors that lead to *inequality*, as well as *cultural understandings*, including attitudes, values, beliefs, and behaviors that may have inter- and intra-cultural variance (Cliff et al., 2009). Indeed, in our work we have conceptualized pedagogies for social justice as “teaching practices that assist students to examine and challenge the status quo, the dominant constructions of reality and the power relations that produce inequities, in ways that can lead to advocacy and community action” (Wright, 2004 p. 7). So why do we use the term “pedagogies for social justice” rather than the perhaps more common term “critical pedagogy” (Lather, 1998)? Whereas some see critical pedagogy to be necessarily radical in nature and as such, seek to critique, disrupt and transform existing social structures that support societal inequities and injustices, others focus more on the class environment and possible issues of inequity and injustice that exist within this context. For this reason we have chosen to use of the term “pedagogies for social justice” which we view to be the teachers' practices that seek to address a broad-brush of inequities and social injustices of significance to HPE through both challenging structures that cause inequities (e.g., gender inequities; racism) but also addressing inequities caused by these structures (e.g., economic disparities). In this regard, it is not dissimilar and is intended to capture Kirk (2020) description of critical pedagogy in the context of HPE, as being “concerned with the organization and alignment of curriculum, teaching, learning, and assessment in ways that render physical education inclusive, fair, and equitable as an embodied experience for young people” (p. 101).

Following the example of Shelley and McCuaig (2018), who initially drew from Gore (1998), we also noted that the call for pedagogies for social justice in HPE has been, “strong on social vision … but weak in terms of classroom or instructional practices” (Gore, cited in Shelley and McCuaig, 2018, p. 513). As Shelley and McCuaig (2018) state, 20 years on from Gore's (1998) challenge to “translate their visions into practice” (p. 274), little has changed.

In this paper, we present nine examples of pedagogies for social justice that we believe both address and act on social inequities which in turn can greatly contribute to the physical, social, and emotional wellbeing of young people. In the next section, we provide an account of the methodology used to generate the data upon which these pedagogies for social justice are based.

## Methodology

In the EDUHEALTH project we employed a “bottom up” approach by focusing directly on the teachers' pedagogy as we wanted to identify the teachers' actions that addressed social justice and the thought processes associated with these actions. The research was informed by Critical Incident Technique (CIT) methodology, a qualitative research methodology that was developed as a way of identifying the significant factors that contributed to the success or failure of a particular event or practice (Flanagan, 1954). Data were gathered from the observation and recording of critical incidents linked to social justice and through semi-structured, post-observation interviews where we explored the thinking behind the recorded incidents. Observations were recorded on a template that identified examples of critical incidents such as practices of inclusion, reflection, consciousness raising, instruction about oppression, prejudice, and inequity that may relate to gender equity (Dowling, 2009), racism (Fitzpatrick, 2013), democratic rights (Dover, 2013), motor elitism (Hunter, 2004), and interpretations of bodies (Tinning, 2010). Participants were 13 HPE teachers in Sweden (4), Norway (3), and New Zealand (6). The 13 teachers were known by at least one member of the research team and were selected through purposive sampling (Bryman, 2016) as “good” examples of teachers who attempt to foreground social justice in their teaching practice. All teacher names referred to in this paper are pseudonyms.

This brief outline belies the iterative process of designing the research project. One of the initial phases of research project included visits to schools in the country of the “other” EDUHEALTH researchers and piloting of the observation and interview template. These included visits to primary and secondary schools, an outdoor education class outside the normal school boundaries, rural, and urban schools, a private school, a boys-only school, and a new school with a purpose built modern-learning environment. Through the eyes of the “outsiders,” we were able to see how practices and structures within each context served to reduce (or attempt to) reduce some of inequities that impact on wellbeing. This included aspects of indigenous Māori culture and language that were evident in schools and infused in the practices of some teachers in Aotearoa New Zealand and the social-democratic practices of Sweden and Norway, such as the provision of school lunches in Sweden, the non-hierarchical clothing of teachers, and reference by first name rather than surname. While we do not report formally on these observations in our study findings, these experiences heavily influenced our understanding of how different cultural contexts frame our understanding of social justice in HPE (Schenker et al., 2019) and the enactment of social justice in HPE practice (Linnér et al., 2020).

During these seminal observations we came to recognize how our own life histories, particularly our previous and ongoing roles as teacher educators with backgrounds of observing student teachers served a valuable role in helping understand classroom practice, but also required us to recalibrate our focus from a broad conception of quality teaching to a narrower focus on pedagogies for social justice. The pilot observations thus served to develop a level of skill and observer expertise (Cope et al., 2015) as “critical incident observers” of pedagogies for social justice in HPE. With respect to our life histories, our initial forays into schools also highlighted the need to recognize and respect the theoretical and culturally located frames of reference that lead to researchers' interpretations of social justice issues in HPE. We may have underestimated the challenge of understanding and accepting each other's position regarding what matters most in the name of social justice and equity. Reflexively, we recognized that rather than positioning the culturally located frames of reference as problematic, we endeavored to embrace the outsider perspectives as being integral to the success of the project because they disrupted our taken-for-granted perspectives of the insiders (Gerdin et al., 2019).

A six phase thematic analysis approach (Braun and Clarke, 2006, 2013) to draw out themes that were important to the research questions. As a first step, researchers in each of the three countries (Aotearoa New Zealand, Norway, and Sweden) read and coded the observation notes and interview transcripts separately. In the second phase of analysis, researcher pairs from each country then met to compare, cross-check, and reduce initial codes and themes into common/shared codes and themes. The third and final level of analysis was a group analysis by all members of the research team *via* two online video conferences (for a full description of the study design see Philpot et al., 2020). In summary, the three final themes (i) “Building relationships,” (ii) “Teaching for social cohesion,” and (iii) “Explicitly teaching about and acting on social inequities” we have reported previously (Gerdin et al., 2020) were generated through a 3-year iterative process that required us to examine our own assumptions, our own culturally located taken-for-granted practices, and our methods of defining what we were looking for and how we went about finding it in a systematic way.

Based on these themes and as way of offering practical examples of implications for HPE practice, we curated nine *pedagogies for social justice* that are substantiated in this paper with the original data generated from interviews and observations. Our construction of these pedagogies was importantly informed by a continuum of theories of, and pedagogies for, social justice from both within and away from the field of HPE. These ranged from humanistic education (Maslow, 1943) and the seminal work of PE scholar Don Hellison's (2011) Teaching for Personal and Social Responsibility (TPSR) model; to post structural and critical theory (Hooks, 1994; Ladson-Billings, 1995); to critical and transformative pedagogy (Freire, 1970; Tinning, 2002, 2017; Ukpoduku, 2009). In drawing on this big tent of social justice theories (Lather, 1998), we want to emphasize that pedagogies for social justice should aim to both address individuals needs and challenge and seek to transform social inequities to improve both student and societal wellbeing in HPE.

## Findings

### Nine Pedagogies for Social Justice

**Figure 1:**
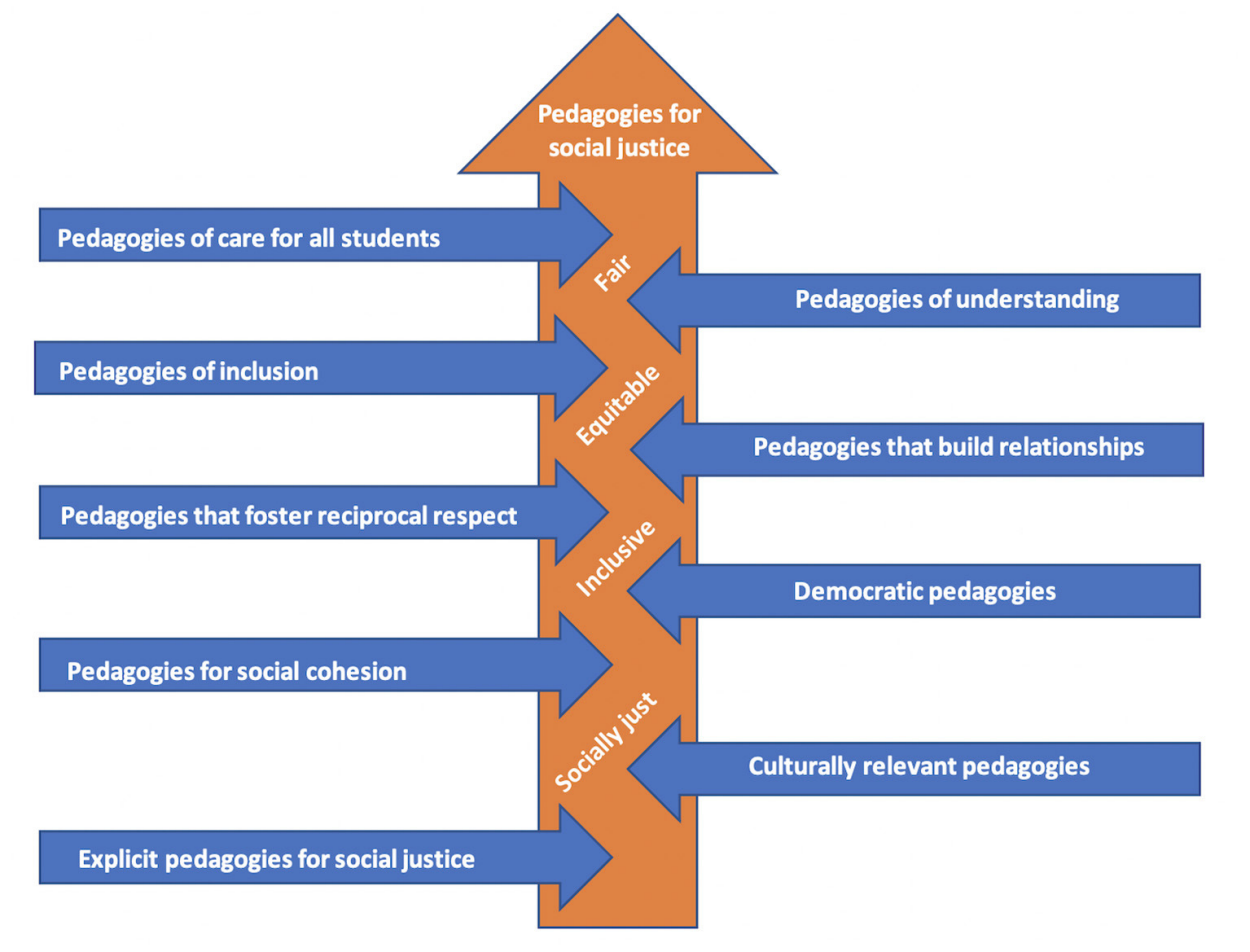
Nine pedagogies for social justice.

In the following findings sections, we provide specific examples of the EDUHEALTH participant-teachers' practices that we consider to be pedagogies for social justice that we observed in the gym, courts, and fields; the interactive practical spaces of HPE. This is the contextual nature of embodied learning in school HPE. We begin with a figure (see Figure 1 below) that is generated from the study data and follow this with more detailed descriptions and specific examples that reflect what we observed. We have taken these specific examples directly from the quotes of the participant-teachers or our own observations notes. Teachers who use pedagogies that have social justice intentions, often use.

#### Pedagogies of Care

Two common characteristics of the participant-teachers in the EDUHEALTH project were their caring dispositions and their genuine concern for the wellbeing of their students. Although these teacher characteristics include practices of being “nice” to their students, a pedagogy of care involves much more than this. Pedagogies of care include teachers' actions that show they genuinely care about the inclusion of all students, the nature of the relationships within the class, and the depth of emotions being expressed in multiple ways by students (Table 1).

**Table 1:** Pedagogies of care.

**Observation**	**Interview**
• The teacher met the students in the center and welcomed everybody to the gym. Some of the students gave the teacher a hug and some just walked in (Emma, SWE) • The teacher used touching (appropriately). She touched one student on the head and one on the arm and some students gave her a hug when they arrived at the gym. The teacher tried to stop a struggle and hugged the boy that was most angry and talked with him (Charlie, SWE) • The teacher used a relatively informal non-confrontational style with the class with the aim being to show a caring supportive approach and build a cooperative collaborative class environment (Louise, SWE). • She described herself as being a caring mother figure as much as a teacher who is giving sound, experienced and knowledgeable guidance to her students (as in a form of ownership and responsibility for their wellbeing) (Candice, NZ)	• “I always try to avoid people feeling like they are exposed” (Emma, SWE) • “I think it is very important for them to feel safe” (Kari, NOR) • “I just want to make them feel comfortable” (Louise, SWE) • “I feel like a bit of a mum quite a lot with them and they need to hear positive things from us” (Candice, NZ) • “I looked at these kids and went wow…some of these students don“t have structured parents…I felt it was something that was almost like combining a little bit of parenting with a little bit of teaching” (Kendall, NZ)

Although many of the teachers did not overtly acknowledge it as such, their caring disposition, and prioritizing of their students' wellbeing above all other factors was implicit in laying a foundation for pedagogies for social justice. Whereas, some teachers explicitly focused on pedagogies for social justice in their practice, others aimed to achieve the same goals without them even consciously conceptualizing their practices as pedagogies for social justice.

The caring teachers we observed used their knowledge about their students, together with reflection, and caring teaching strategies to arrange educational environments that could lead to more equitable and socially just outcomes for all students. When used in this way, a *pedagogy of care* is a crucial element in laying a foundation for pedagogies for social justice in HPE. However, it must be said, that a pedagogy of care does not automatically result in pedagogies for social justice. For example, a pedagogy of care that focuses *exclusively* on making students comfortable cannot be considered a pedagogy for social justice unless the teacher has a further intention of addressing issues of equity and social justice. A pedagogy of care sits at the heart of social justice pedagogies (Fitzpatrick, 2013, Lynch and Curtner-Smith, 2019) but it is only a foundation for social justice when it builds the trust needed to engage in “pedagogies of discomfort” (Shelley and McCuaig, 2018, p. 517) that disrupt taken-for-granted practices and act on social inequities.

A pedagogy of care is shown in the dispositions and actions of the teachers when they show that they genuinely care about their students' emotional and social wellbeing. Drawing on Nel Noddings' care theory (1984, 1997), we argue that these caring teachers use their knowledge about their students, together with reflection and caring teaching strategies, to create a learning environment that promotes inclusion and equitable outcomes for all students. That is, to act “as one-caring, is to act with special regard for the particular person in a concrete situation” (Noddings, 1984, p. 24).

#### Pedagogies of Understanding

A *pedagogy of understanding* is an extension of a pedagogy of care in that it requires teachers to seek to understand their students' particular circumstances and needs. Again, this draws on and is supported by Noddings' (1984, 1997) care theory. In this way, a pedagogy of understanding can only begin when teachers take action to get to know their students and their individual circumstances, and care enough to create a classroom environment that is conducive to learning. Such a pedagogy starts with the assumption that equity is about treating individual students differently because of their different circumstances and needs (Table 2).

**Table 2:** Pedagogies of understanding.

**Observation**	**Interview**
• The teacher asked some students to swim in different ways and to stay in the pool to ensure that the students who were slow were not under the gaze of other students (Emma, SWE) • There was one new immigrant girl wearing a headscarf [hijab] in the class. When the children got into the pool to swim the lengths she swum two lengths but was not too confident so got out and sat on the side. The teacher went to her and spoke to her in an understanding manner (Emma, SWE) • Some students arrive late. The teacher welcomes them and carries on with the lesson.The teacher later explains that the students who were late are often the students who don't have bikes or mopeds. He doesn't think it is fair to penalize them for their lateness (Kane, SWE) • Those who did not have HPE clothes were not pointed out and they were allowed to participate (Tane, NZ) • One of the boys was not changed for HPE but rather than confront this and make it an issue the teacher accepted it and seemingly would rather have him participate than sit out (Charlie, SWE) • One student has no shoes, so she gets the teacher's keys to pick up and borrow a pair from the teacher (Louise, SWE)	• “I could tell she was a bit hesitant about it, so I stood next to her and went 3, 2, 1 let”s go together and she did it and I did it” (Gary, NZ) • “One of the boys didn't have HPE gear today and he was a bit flustered. I could have told him off, but I could see that something had happened… so I just went and found him gear. In that situation, it is a case of just trying to have empathy with them and just maybe understanding that something has happened” (Candice, NZ) • “I think maybe their parents can't afford to buy them what is needed, and I take that into consideration” (Dillon, NZ) • “Some don't have HPE gear just because of the world they are navigating at home and I know that” (Gary, NZ) • “I have optional clothing for swimming” (Emma, SWE) • “First and foremost, I want them to be able to participate and then their uniform is something that we have conversations about later” (Gary, NZ)

During their interviews, it was very common to hear the participant-teachers say something like “I know that student and I know his or her background (perhaps family background) or particular circumstances so I take that in account when dealing with him or her.” We saw examples of the teachers' pedagogies of understanding when some students were late for class, or new to the school, or could not afford HPE uniforms or were obviously upset perhaps because they we having hard times at home or elsewhere, or just because they did not like HPE for some reason. In each case, the participant-teachers showed that *they tried to understand the particular circumstances* of the student. For example, in one class when two boys began to fight, the teacher stopped the fight and then hugged the main offender to calm him down and let him know that it was okay. There was no further punishment because the teacher knew his circumstances and understood that he needed this personal attention and care. She knew that this was the best way to support both his wellbeing and his learning.

In another example, several students arrived late for class even after the initial introduction to the lesson. The teacher did not question or punish them for lateness. In the follow up interview, the teacher explained that the late students did not have transport and had walk to the track whereas the early-arrivers had bikes or mopeds, so he simply welcomed the late comers and, after explaining what they needed to do in the lesson, he carried on with the lesson, showing his understanding and sound reasoning. Typically, pedagogies of understanding involve on the spot, subjective teacher decisions with regards to attendance, lateness, HPE uniform or participation based on the teacher's knowledge of individual student circumstances.

#### Pedagogies of Inclusion

Pedagogies of inclusion are a further extension of the teachers' pedagogies of care and understanding. They are an extension in that they require a further step of not only knowing and caring about all students but also taking steps to make sure that all students are included. In this EDUHEALTH project, the teachers showed that a pedagogy of inclusion was particularly important when the teacher knew that there was potential for a student or group of students to be excluded for one reason or another. Therefore, their pedagogy of inclusion was often pre-emptive rather than reactive. The genesis for such an approach was typically a teacher-perceived concern about the class or different group dynamics within the class, which could be the result of ethnic, religious, gender, sexuality, nationality, language, physical ability, and/or cultural difference (Table 3).

**Table 3:** Pedagogies of inclusion.

**Observation**	**Interview**
• She welcomed the students in a very friendly way, established a positive climate and promoted a sense of belonging (Kari, NOR) • The teacher was aware of this boy's needs and went to lengths to ensure that he was included (Emma, SWE) • The teacher and students sit in a circle on the gym floor to discuss objectives, the nature of games and why they are playing them. This allows everybody to become involved in the discussion. It is an inclusive pedagogical technique (Kari, NOR) • At the beginning of the lesson one boy does not have a bike. It appears that all the others do. A teacher or teacher aid goes to the basement and gets one for him, so that he doesn't miss out (Emma, SWE)	• “I use strategies like pairing new students with others in the class” (Emma, SWE) • “I adapt my way of teaching to include new immigrant children by using different forms of inclusive body language” (Kane, SWE) • “I group them based on who I think they would be most comfortable with” (Emma, SWE) • “It is not fair sometimes that they haven't had the same opportunities that others have had and if I can be part of the big picture that helps those students to achieve closer to their potential, then that is what I want to do” (Kendall, NZ) • “I look at the teams and if I see that a team is struggling a little bit, I jump into that team just to help them out” (Candice, NZ) • “I think that sitting down with them and being positive toward them is important. Positivity is the thing that is going to have people enjoy this [HPE] more and feel included” (John, NZ) • “I have a student in 9th grade. HPE is not her subject, but she loves to shoot, and I know that there is a shooting place next to the hockey arena. So, I said why don't we go and shoot for a lesson, and she said, yes sure. So, we took the whole class up there and she was able to shine in front of the whole group in something that is her subject. She is normally very shy and doesn't want to take a place in a group, but she could go in there with confidence and feel, I know this, and instruct the whole class” (Kane, SWE)

Pedagogies of inclusion usually begin by recognizing the potential for individuals or groups to be marginalized. This, once again, requires teachers to know their students and be aware of their specific needs, particularly those of the marginalized students or marginalized groups. However, for many teachers, the aim of pedagogies of inclusion is often simply to include all individuals and build a harmonious class environment, but this is not necessarily introducing pedagogies of social justice. For it to become a pedagogy for social justice, the pedagogy requires teachers to recognize and address the reasons for non-inclusion or the practices of exclusion and marginalization and this means addressing it with both those who marginalize (sometimes unknowingly) and those who are marginalized. Drawing on Noddings again, we argue that this requires teachers to *listen* to the students, *engage in dialogue* about marginalization with them, *draw connections to life* beyond the HPE classroom for these students, *think critically* about the underpinning reasons for exclusion and then *reflect and respond* using inclusive practices (Nodding, 2012).

A typical example within a HPE class is the marginalization of new immigrant students, or students who are different from the norm, students who are less physically able or the marginalization of girls when playing mixed invasion ball games. Such discriminatory occasions are ideal “teaching moments” for addressing inequities and injustices in a context that the students can understand and reflect upon because it is a situation that is real and specific to them. That is, these moments lend themselves to enact pedagogies of inclusion that can be deemed to be pedagogies for social justice, when the teacher perhaps asks—what is happening in this game? Who is benefitting and who is not? How can we make the situation fair for all? Who wins and who loses when we play it the way we are? Is this fair? How can we make it more inclusive? We can also take a further step and ask how the exclusion practices and inequities that often go unnoticed in our class reflect the way we act as a community or society. How does stereotyping and normalizing privilege and discriminate? We can see that this pedagogy takes planning and usually pre-lesson consideration of the “potential teaching moments” and the actions required by the teacher. Inclusive pedagogy, then, often begins by identifying exclusiveness (or the potential for exclusion) and its impact on particular students, and then the planned, enactment of inclusive practices.

#### Pedagogies That Build Relationships

Almost all of the participant-teachers placed high importance on building relationships between themselves and students, and students and other students. In the EDUHEALTH project we found examples of teachers getting involved in the games or taking the time to talk one-on-one with their students as they walked to the gym or sitting with them to talk at their level and asking inquiring questions to get to know them. We also saw several well-planned lessons in which the students were intentionally grouped together in cooperative game activities so that they could get to know one another better. In some classes we saw incidental examples of students engaging in relationship building unprompted by the teacher, for example we saw one student who cared about and befriended a new student. They biked to the swimming pool together at the back of the class-group and swam together with the first showing the second how to dive off the side of the pool. Within the theme of building relationships, we identified three subthemes that can lead to improved relationships; (1) knowing the student(s), (2) reflecting on individual, environmental and relational aspects, and (3) using caring teaching strategies (for a full discussion of caring teaching and building relationships see Mordal Moen et al., 2020). Well-being can be seen to be at the forefront of pedagogical work that uses learning activities that are inclusive and conducive to relationship building and create environments that breakdown social structures that create social exclusion. Inclusion is of critical importance as friendships and social inclusion are known to be protective of wellbeing (Wilkinson and Pickett, 2010) (Table 4).

**Table 4:** Pedagogies that build relationships.

**Observation**	**Interview**
• When asked about her interactions with the students the teacher said, “they need positive interactions—they really want to please us” and “they want to do well” (Kari, NOR) • The teacher has individual chats, before the lesson and after the lesson. He describes it as an important tool to build relations (Ola, NOR) • The lesson starts before the students enter the room (with individual chats) and ends after the students have left (Charlie, SWE) • The teacher's relationship with the students was important in establishing an empathetic learning environment (Tane, NZ) • Well planned progressive lesson with an obvious objective of developing collaboration and cooperation—Although we didn't see the planning it was obvious from the content that the teacher had thought about the nature of the activities and planned the progressions with the intention of requiring the students to integrate with different others and work collaboratively in different pairs and groups (Kari, NOR)	• “I have a relationship with my students, where I feel like a bit of a mum quite a lot with them and they need to hear positive things from me” (Kendall, NZ) • “I find it almost impossible to teach if I don“t have a relationship with the students” (Candice, NZ) • “I am not an authoritarian leader at all. I am quite comfortable with stepping back and letting them do it, but you can only do that if you have got the relationship with them” (Ola, NOR) • “We played a really boring name game, and it was actually perfect because now I know all of their names, it makes a big difference” (Louise, SWE) • “I know a lot of their older brothers and sisters, which has actually helped as well because they can relate to me” (Candice, NZ) • “One of the boys can be a bit of a handful but he is fine with me… I just take the time to talk to him like when we are walking up and walking down (to the gym)” (Candice, NZ) • “We have worked on, call it team building activities. It is the second year I have them [this class]…we have worked a lot with activities to avoid such unintended conflicts between groups in the class” (Per, NOR)

#### Pedagogies that Foster Reciprocal Respect

When they were interacting with their students, most of the participant-teachers treated the students with care and respect, which was befitting of a humanist pedagogy of care. These teachers recognized that each person has the human right to be treated with respect when in the shared social space of the class environment (albeit in different roles). It is important to state that this was not just typical classroom teacher pedagogy where the teacher asserted their position of power and the students were respectful of the teacher because of this position. What we observed was relationships that were less hierarchical and less teacher directed, and more collaborative and dialogical (Freire, 1970). The teachers appeared to seek talk with students when addressing typical classroom management issues such as organizing groups, dealing with lateness and off-task behavior. The body language of students suggested that they were actively involved in working toward a resolution. It is what we came to call *a pedagogy of reciprocal respect*. There was an inherent expectation that if the teacher is respectful to the students and sets the standards for the students then the students will return that respect in kind. This is more than mutual respect, which is rather passive and benign in nature. Reciprocation inherently means giving and receiving back. With this understanding, we observed that reciprocal respect was given and received through respectful and supportive interactions between teachers and students. The most important learning to come from this, for some of us, particularly those of us who practice in the Anglocentric world, was the noticeable difference between a pedagogy of reciprocal respect and that of authoritarian control, which is a form of dominating pedagogy so often characterized by the “sporty-male” HPE teacher. A pedagogy of reciprocal respect moves students beyond engaging in learning activities out of respect for the teacher authority and within teacher designed classroom structures toward a position of more agency where they have the responsibility to make decisions within the classrooms (Table 5).

**Table 5:** Pedagogies that foster reciprocal respect.

**Observation**	**Interview**
• The teacher encouraged the students to be respectful of each other to build relationships with and between the students (Charlie, SWE) • The teacher said he was a positive person who always wanted to present a positive attitude in the hope and expectation that this would be modeled by the students (John, NZ) • The teacher smiles and laughs and tells jokes with the students. She laughs. They laugh (Kari, NOR) • The teacher uses a relatively informal non-confrontational style with the class (Louise, SWE) • The disposition of this teacher establishes a positive role model for the students to follow. It is difficult to image how the students could not be inspired by the positive attitude. It is infectious and helps to ensure the students engage in the activities with the same level of enthusiasm and enjoyment (Kari, NOR) • We have seen 10 teachers so far. All of these teachers speak quietly (but at times assertively) to the students. There is no yelling and no public confrontation (e.g. for being late, incorrect uniform) (Emma, SWE)	• “They are really respectful. I guess it is that I try to like them, and I think they can pick up on that. If they know you like them, they respond well to that. Nobody likes to talk to somebody who they feel doesn't like them” (Candice, NZ) • “I think it's very important that all students are heard. They have respect fellow students, so I tend to be very strict about it” (Ola, NOR) • “I want to be a teacher who is not in their face yelling at them. I just want to be calm and I want them to understand where I am coming from” (Dillon, NZ) • “I believe that we have PE with boys and girls together to work together and respect each other and respect each other's differences” (Kane, SWE) • “If we want them to learn, then we need their environment to be really good or amazing for them” (Dillon, NZ) • “I think when someone is positive to you, you are inclined to be quite positive yourself and you feel as if you have done something well and it sets the tone” (John, NZ) • “It is not just teaching wise, it is an interaction, as a social thing. I think it is really important to start with something positive” (John, NZ)

#### Democratic Pedagogies

The democratic process is central to building fairness, trust, and voice at all levels of society. Many of the participant-teachers demonstrated their use of democratic pedagogies by giving students choice and/or options. It was a pedagogical approach in which both students and the teacher had a say in content, game rules, or teams etc (see e.g., Butler, 2021). This approach is about power sharing or making explicit that they, the students, have a right to a view and a voice in the class as well as the teacher. It begins by recognizing that all students have a democratic right to ask questions and/or challenge others' decisions if they believe learning activities are not fair or because they are being excluded in games. It continues by encouraging and empowering students to do so in a considered manner. Strategies for including student voice and sharing power show a willingness on the part of teachers for their lessons to be less teacher directed. In implicit and explicit ways, sharing power within HPE settings is an effective means of introducing practices that address inequities and social injustices (Oliver and Kirk, 2016; Luguetti et al., 2019) (Table 6).

**Table 6:** Democratic pedagogies.

**Observation**	**Interview**
• The teacher seemed to spread the questions around by calling on different students for answers. She also asked the students to come up with ideas of how they could improve the cooperation by changing the rules, for example (Charlie, SWE) • When the students are encouraged to talk in front of the class he sits on the floor, implicitly demanding the other students to do the same. He tells us that it is very important that no one interrupt the student who is speaking. It is about respect (Ola, NOR) • When the teacher introduced the theme for the lesson, she was very enthusiastic and let everyone be involved in the discussions (Kari, NOR) • The teacher put together groups based on the students' assessment of their own level of ability from 1–10. The students adapted the rules of the game in order to make the game fair for all (John, NZ) • During the games when someone cheated and others complained to her, she didn't respond but rather afterwards said how did you feel when they cheated? How did it affect the game and what shall we (collectively) do about it in the next game? The expectation was that the students themselves (with her support) would come up with the solutions to their issues rather than her (Kendall, NZ)	• “I get down to their level. They still know I am the teacher, but I am more approachable, they are more open and there is a lot more discussion” (Dillon, NZ) • “It is a fine line, but it is okay for me to have a bit of a joke. It just takes away that authoritarian approach” (Dillon, NZ) • “After playing the game for part of the time, I asked the students, if we should take that rule away for the final game and they agreed that we should be fair to everybody” (Tane, NZ) • “I cannot stand there and work from the top down the whole time… just by bending down I can change the balance of power a little. If we want to communicate more on an equal footing and hear what kind of experience they have had, reflections they have, then it is natural that I sit at the same level” (Ola, NOR) • “We spent a lesson talking about how we could provide opportunities for as many pupils to participate and demonstrate skills as possible. These [the game rules] are all their ideas. They came up with the idea of giving themselves a ranking, and they came up with making teams based on averaging out the numbers. So, that is completely the students' idea” (John, NZ)

Generally, the participant-teachers' pedagogies took one or both of two forms, firstly some took action to reduce the student-perception of teacher power and dominance and secondly, many used student choice or student input to provide student voice and empower the students. In the first instance, many participant-teachers used non-authoritarian approaches, by engaging in conversation or discussion rather than didactic-instructional modes, some lowered themselves to sit at the same level as the students. Others played games with the students, while still others included the students in the actual lesson planning. Many of these involved the teachers and students engaging in examination and discussion about fair play, fairness within the class, and self and peer management.

#### Pedagogies for Social Cohesion

Teaching for social cohesion is a pedagogy that requires teachers to proactively address the nature of the interactions between individuals in heterogeneous groups. Just as the physically interactive, movement contexts of HPE offers an excellent medium for building relationships between students it offers the opportunity to take a further step and build social cohesion at a more group community or societal level. This requires the expertise of competent teachers. The apparent need to teach acceptable social behaviors and develop cooperation and group cohesion that enable positive class learning environments was a common theme among the participant-teachers. Teaching for social cohesion also became the second of the three themes of the EDUHEALTH project. However, the differences between building relationships, teaching for cooperation and teaching for social cohesion requires deeper explanation. At times this was not so obvious in our observations of the participant-teachers' pedagogy. Allport's (1954) contact theory puts emphasis on the value of deep, meaningful contact between different individuals and groups for developing an understanding of the other and in this way provides a platform for developing social cohesion. Pettigrew's (1998) extension of Allport's theory also includes group cohesion in which he details essential elements for addressing differences between in-groups and out-groups, which we often identify as being dominant and marginalized groups. The essential element for social cohesion involves recognizing and accepting differences and then finding ways to work together to be inclusive and fair to all. For a more in-depth explanation of social cohesion and pedagogies for social cohesion see Smith et al. (2021) (Table 7).

**Table 7:** Pedagogies for social cohesion.

**Observation**	**Interview**
• When grouping the students the teacher seeks a mix between gender and ability and also to avoid the students always being together with their closest friend/s (Charlie, SWE) • The teacher was conscious of the need to ensure that they were mixed up to play with others they do not normally play with. Again, with the ultimate agenda being to enhance social cohesion (Kari, NOR) • The apparent need to teach group cohesion and social behaviors that are respectful and caring of others as well as enable collective thinking, decision-making, and acting for the good of all, seems to be a first step for these students (Charlie, SWE) • Social cohesion and building social responsibility overrode all other content objectives to the point that the nature of the activity (Turbo touch) was just a known and enjoyable medium for teaching the more important “bigger matters” of establishing strong social cohesive values (Candice, NZ) • The teacher introduced the lesson by explicitly showing that the aim was to develop collaboration She used a card with the word collaboration on it so that the students could clearly see that this was the lesson focus (Charlie, SWE)	• “I think the outcome is social integration and feeling confident as part of a group or as an individual and how you can contribute to the environment around you” (John, NZ) • “The groups are mixed gender and I put them into their specific groups on purpose” (Kendall, NZ) • “I do force some processes…they have to work with someone they usually do not communicate much with to build relationships with each other” (Ola, NOR). • “One of the reasons that we have HPE with boys and girls together is because we are supposed to be able to work together and respect each other and respect each other's differences” (Kane, SWE) • “It is having a balance, sometimes you are going to work with your friends and sometimes you are going to collaborate with people who you might not even like, but you need to” (Gary, NZ) • “They have to take part in group processes, and they have to work together, and they have to discuss how they work together as a group and whether this is successful or not” (Gary, NZ) • “When you see the makeup of culture and gender you again come back to the whole society thing and fitting in with people and not discriminating based on them being different” (John, NZ) • “The idea was to have them working in groups, working toward a common goal focusing on interpersonal skills. There were five or six interpersonal skills we looked at as a class, but they only chose three” (Dillon, NZ)

In all three countries of the EDUHEALTH project the participant-teachers perceived that their students needed to learn how to work cooperatively and constructively with others. Our observations would suggest that teaching *for* social cohesion is potentially one of the most important social outcomes of compulsory HPE and potentially a precursor to improved physical, emotional and social health outcomes when it becomes an explicit focus. This is particularly so in school communities that are characterized by diverse socio-economic, ethnic, cultural, gendered, and religious backgrounds. Teaching for social cohesion is not just about having students cooperate or work together in groups; it is about using these groupings to build understanding and acceptance of differences and learn to appreciate the need to live and work as a community or society with others who are different from you or may have different values and beliefs than you have.

#### Culturally Relevant Pedagogies

Culturally relevant pedagogy provides a way for students to maintain their cultural identity and integrity while succeeding academically (Ladson-Billings, 1995). Klug and Whitfield (2003) later suggested that culturally relevant pedagogy is teaching that is done in a way “that ‘makes sense' to students who are not assimilated into the dominant culture” (p. 151) (Table 8).

**Table 8:** Culturally relevant pedagogies.

**Observation**	**Interview**
• The teacher used te reo Māori in the chart and on the whiteboard. “He mahi tahi tatou mo te oranga o te katoa—we should all work together for the wellbeing of everyone.” This was also evident in her use of Hellison's five levels. The levels were given Māori names with the top one being manaakitanga—(respect and caring), which was also one of the school's values (Kendall, NZ) • The teacher recognized and incorporated indigenous and other marginalized languages and cultural values in his teaching (Dillon, NZ) • He says that his early teaching experiences with exclusively migrant children have reshaped his thinking about the purposes of physical education. He discusses many strategies and incidents. He has learned to communicate in many languages. He stressed to us the power of using the language of migrant students and teaching them the Swedish language to ensure that they can succeed in the Swedish system (Kane, SWE)	• “In Sweden it is compulsory for all students to be able to swim 200m. We have a lot of Muslim girls who didn't want to swim with boys so every other week on a Friday we take the Muslim students to the pool and we had girls only and then it is boys only. Then we had the Iman from the mosque come here to talk to them and he said that it is okay to swim together if the girls have a full body swimsuit that covers their hair. So now they can participate” (Kane, SWE) • “We are doing a big project. It is culturally responsive relational pedagogy that is across the entire staff…it is looking for opportunities to be able to incorporate any type of tikanga or Māori culture into our teaching” (Kendall, NZ) • “I use Māori tikanga [protocols] and language to reflect my commitment to Te Tiriti o Waitangi” (Kendall, NZ) • “We use an interpreter [to communicate with families] and sometimes we use older siblings if it is about less important things like to ask them to bring clothes. We use translators to send written information.I really do not want to use someone else, but language is a big challenge” (Charlie, SWE)

The majority of the EDUHEALTH participant-teachers recognized cultural, religious, and gender differences and often responded by applying different forms of pedagogy for different groups. Some teachers actively sought to connect with recognized leaders or authorities in the local community that had authorized powers to represent the interests of different ethnic or religious groups. These were leaders who were able and willing to share their knowledge of values and practices that could be adopted in class and school. For one of the Swedish teachers this involved meeting with the local Imam, to discuss the Muslim students' participation in swimming lessons. In Aotearoa New Zealand, this could involve local kaumatua (elders) for Māori or church leaders for Pasifika students.

In all three societies the participant-teachers aimed to be inclusive of student of difference and this often meant that they needed to recognize and adapt their lessons to cater for the different needs of these students. Some did this by recognizing the students' socio-economic circumstances, or religious beliefs and practices, while others recognized language difficulties or ethnic values and protocols and, in some instances, used the students' different languages to acknowledge their cultural identities. Most often, this required the teachers to be well versed in the ways of their students and show empathy and understanding even though, for the most part, the teachers did not share the same backgrounds as the students. In some cases, it meant proactively seeking to address exclusion, marginalization or non-recognition of what mattered most to these students. To reiterate, it is our view that culturally relevant pedagogies have the potential to, in particular, improve the physical, emotional, and social wellbeing of students who are not members of the dominant culture.

#### Explicit Pedagogies for Social Justice

Freire (1970) stated that critical pedagogies, or what we term in this paper, pedagogies for social justice, are practices whereby teachers reflect and act on their world in order to change it. Explicit pedagogies for social justice, actively seek to address social justice issues as the learning content or the outcome of a lesson. This often involves addressing issues of identity positioning that define, differentiate and discriminate against minority groups. They are explicit attempts to actively make these issues explicit in HPE lessons and specifically aim to address issues such as racial stereotyping and racial discrimination, gender, and sexuality discrimination, nationality and religious discrimination and physicality (body shape) and ability discrimination. These are issues that are all too common in physical education and often reinforced rather than challenged by the actions or inactions of physical education teachers (Ennis, 1999; Fitzgerald, 2005; Stride et al., 2020) (Table 9).

**Table 9:** Explicit pedagogies for social justice.

**Observation**	**Interview**
• The teacher tries to touch on what is meaningful for each student at the individual level. He wants the students to reflect on their feelings. He really tries in the next step to get the students to reflect on sports culture, gender issues, etc. and if that affects their experiences (Gary, NZ) • The teacher said that she actively addresses the negative impacts of past (and current) wrongs, such as colonization, or sexual, gender, racial, and religious discrimination, by explicitly integrating the issues as teaching content with the intention of educating for social justice and equity in society (Kendall, NZ) • The context for the lesson is a lake about 500M from the school. The context is Canadian canoeing. There are approximately 15 canoes and a lock up with paddles and lifejacket. It must have taken a lot of effort to gather these resources (Per, NOR) • The lesson agenda was a hybrid-version of Hellison”s personal and social responsibility levels using a Māori-perspective, traceable in the NZ HPE curriculum. The teacher started by talking about Manaakitanga, that is a Maori-word for caring (as I recall). When explaining her use of Māori culture and Maori-words the teacher said, “I am looking for opportunity to incorporate Māori culture in education” (Kendall, NZ)	• “We want to challenge their opinions about certain sports and racial stereotypes like rugby is for Māori and Pacific people - that is something we try to challenge” (Gary, NZ) • “It is really about breaking down barriers, challenging gender bias. In health, we talk about sexualities and we then try to apply that in HPE, so we talk about how difficult it may be for a transgender person or someone who is considering changing sex in PE. We discuss how we can make them feel comfortable in that space in HPE” (Gary, NZ) • “The school provides some experiences that they can hardly get at home. I have done this in my spare time … I think is very valuable to contribute to it, and I am dedicated to making physical education very fair…” (Per, NOR) • “We play cultural games. One of their units is cultural games, so one of my classes has done ‘ki o rahi‘, a Māori game” (Candice, NZ) • “I had to adapt my way of teaching. If I just work with the language, I wouldn't reach them as much. I used body language and always show or use other students to show them how to do it…” (Kane, SWE) • “The purpose of using different rules for different players is to provide equal opportunity for each student in the class” (Dillon, NZ) • “I actually split the boys up first because the boys are the more dominant ones, but when I wrote the post-it notes, I actually made sure I didn't write the boys at the top. So, I did that purposely” (Candice, NZ)

Most often, the participant-teachers in the EDUHEALTH project did not describe their practices as explicit pedagogies for social justice, but their endeavors to create social change and to address inequities through challenging stereotypes, normalizing indigenous language and culture, and affirming identity are consistent with the purposes of explicitly teaching for social justice. The explicit pedagogies for social justice that we observed included changing game rules to include students with injuries and differing levels of ability, offering culturally appropriate PE uniform options, creating written resources in an indigenous language, and advocacy for resourcing students who through no fault of their own needed extra support to achieve equitable outcomes. As such, they are teaching approaches that are consistent with claims that HPE is an ideal place for improving the health and wellbeing of children and young people (Doll-Tepper and Scoretz, 2001; Morgan and Bourke, 2008).

Explicit pedagogies for social justice was the third major theme to emerge from the EDUHEALTH project and this theme has been explored in detail elsewhere (see Philpot et al., 2021).

## Conclusions

The nine pedagogical practices described in this paper have been presented in a seemingly progressive fashion to show practical examples of different pedagogical approaches to social justice. However, as stated in the introduction, they are not mutually exclusive and many of the examples from one category overlap and work collectively with some of the other reported pedagogies.

In line with Antonovsky's (1996) definition of salutogenic health, we advocate that the HPE pedagogies described in this paper can contribute to healthier individuals and healthier communities. For some who may focus on the role of HPE in addressing chronic diseases (McKenzie and Lounsbery, 2009, 2013), this may represent a shift in thinking from instrumental and individual notions of health and wellbeing and the types of HPE practices that may contribute to these aims. We recognize the physiological benefits that can be achieved through physical activity in HPE, but we agree with Quennerstedt (2019) who argues that physical education must be relevant, *educative* and conducive to future learning and growth. We also agree that physical activity should remain as a context for learning, but caution that school HPE should not be reduced to a short-term physical activity for “health” (reduction in chronic disease intervention) (Gard, 2014).

We argue that HPE must embrace pedagogies that focus on health as both a shared societal responsibility and an individual responsibility. Importantly, these pedagogies must reflect that learning is a social process and secondly, that sociocultural and economic contexts “afford diverse opportunities to be healthy and to learn healthy lives…” (Quennerstedt et al., 2010, p. 108). In the context of HPE, a salutogenic approach to health and wellbeing is as much about *how* we teach as *what* we teach about in HPE. That is, how can HPE practices “help young people to grow as individuals and democratic citizens….become critical and active transformers of society” (Quennerstedt, 2019, p. 11).

Once again, it is our belief that the nine pedagogies for social justice in this paper are conducive to improving the physical, emotional and social health and wellbeing of students, who in different circumstances, may be “enemies” rather than “friends” of HPE (Evans and Davies, 2017). These pedagogies have the potential to challenge a range of structures that create inequities but equally important, to address inequities caused by these structures. While it is alluring to conclude that pedagogies of care, inclusion, understanding, building relationships and social cohesion must precede approaches that are more radical in nature and address the greater societal issues of inequity and social injustice, it is equally likely that actions that seek to address inequity are also those that demonstrate care for others, understanding of difference, and build trust. In some ways, engagement with these nine pedagogies is a circular path with entry points defined by context, the needs of individuals and the needs of the school community.

As HPE teacher educators and researchers within the field of HPE, we recognize that many of these pedagogies are not new to many physical educators, however, we assert that identifying explicit pedagogies *about* and *for* social justice are necessary for teachers who wish to embrace the social justice agenda in their HPE practices. The intention of the EDUHEALTH project was to add more than further critique of the equity and social justice issues facing society. We did not set out to criticize current HPE practices, or tell HPE teachers what they should not do, but rather to find and describe what teachers are doing and can do in HPE to address society's inequities and injustices, particularly as they affect the wellbeing of their students. The nine pedagogies for social justice represent our understanding of the good teaching practices we have observed.

Following the findings of the project we argue that pedagogies for social justice, in a quest to render physical education that is inclusive, fair, and equitable (Kirk, 2020), can have both elements of humanism (i.e., caring, understanding, cooperative elements) that attend to the needs of students within the structures of each society and also challenge these structures through explicitly naming and acting on inequity (Freire, 1970). It is possible, we argue, to scaffold our HPE pedagogy and our students understanding by laying the foundations and encouraging the students to reflect on and act on social justice issues that affect them. This is even more possible when we help them to recognize the democratic processes that provide them with the agency to address equity issues that impact on their wellbeing and the wellbeing of those around them.

We would further like to stress that these pedagogies for social justice should not be seen as unique to HPE as they can equally be adopted by teachers of other school subjects. There is sufficient scholarship in other learning areas to suggest that social justice can be the focus, for instance, in maths (Buell and Shulman, 2019), social studies (Misco and Shiveley, 2016), science (Barton and Upadhyay, 2010) and music (Allsup and Shieh, 2012). If we recognize this and collaborate across the whole span of the school curriculum, we will be in a better position to recognize and accept that HPE has a complementary contribution to make rather than an unrealistic all-encompassing one (Tinning, 2010). We therefore call for the development of strategies to integrate these nine (and other) pedagogies for social justice across various school subject activities and interactions in school community contexts aimed at holistic school wellbeing to contribute to greater equity and social justice in society. However, we would also like to reaffirm the point that context matters (Tinning, 2010; Linnér et al., 2020: Schenker et al., 2019). Pedagogies for social justice need to be sensitive to both subject matter and socio-cultural context in which they are enacted. The recent influx of refugees in Sweden and the lingering impacts of colonization in New Zealand are two examples in this paper of contextual factors that require context-specific socially just teaching responses. We therefore conclude by calling for more research demonstrating contextualized pedagogies for social justice across other subjects and countries with aim of reducing inequalities and improving student and societal wellbeing.

## Data Availability Statement

The original contributions presented in the study are included in the article, further inquiries can be directed to the corresponding author.

## Author Contributions

GG, RP, WS, KS, KM, LL, SL, and KW: conceptualization, investigation, methodology, and writing—review and editing. GG, RP, and WS: writing—original draft. All authors have read and agreed to the published version of the manuscript.

## Author Disclaimer

The results presented in this article only reflects the authors' views, and the European Union is not responsible for any use that may be made of the information it contains.

## Conflict of Interest

The authors declare that the research was conducted in the absence of any commercial or financial relationships that could be construed as a potential conflict of interest.

## Publisher's Note

All claims expressed in this article are solely those of the authors and do not necessarily represent those of their affiliated organizations, or those of the publisher, the editors and the reviewers. Any product that may be evaluated in this article, or claim that may be made by its manufacturer, is not guaranteed or endorsed by the publisher.
